# Endoscopic Ultrasound Guided Pancreatic Pseudocyst drainage experience at a tertiary care unit

**DOI:** 10.12669/pjms.36.4.1442

**Published:** 2020

**Authors:** Erum Kazim, Muhammad Ali Taj, Imrana Zulfikar, Jawad Azeem

**Affiliations:** 1 Dr. Erum Kazim, FCPS. Department of Surgery, Dow University of Health Sciences, Karachi, Pakistan; 2 Dr. Muhammad Ali Taj, MRCP, Department of Surgery, Dow University of Health Sciences, Karachi, Pakistan; 3 Dr. Imrana Zulfikar, FCPS. Department of Surgery, Dow University of Health Sciences, Karachi, Pakistan; 4 Dr. Jawwad Azeem, SMO. Department of Surgery, Dow University of Health Sciences, Karachi, Pakistan

**Keywords:** Endoscopic ultrasound (EUS) guided drainage, Pancreatic pseudocyst (PPC), Outcome

## Abstract

**Objective:**

To evaluate the clinical success, technical success and complications related to Endoscopic ultrasound (EUS) guided Pancreatic pseudocyst (PPC) drainage.

**Methods:**

This retrospective study was conducted on the patients with symptomatic PPC who presented over a period of three years, between January 2015 and September 2018, at Endoscopic Suite of Surgical Unit 4, Civil Hospital, Karachi. Record was analyzed for demographic data, indications for the procedure, complications and success related to EUS guided drainage. Statistical analyses were performed using the SPSS Version 22.

**Results:**

Total number of patients was 71. Mean age was 37.20 ± 17.27 years with a range of 6 to 68 years. Complications occurred in 12 (8.52%) patients, including stent migration (5/12), bleeding (4/12), infection (1/12), intra-abdominal abscess (1/12) and perforation (1/12). Technical success was achieved in 100% and clinical success in 97.1%. There was no procedure-related mortality.

**Conclusion:**

Pancreatic pseudocyst (PPC) is a known complication of acute as well as chronic pancreatitis which can have dreaded and appalling effects. In this part of the world with limited and scarce resources, EUS guided drainage of PPC is most feasible and rational with minimal complications, thus making it a front runner procedure.

## INTRODUCTION

Endosonography has now become the spearhead treatment modality in acute or chronic post pancreatitis complications, commonly known as pancreatic fluid collections (PFC), enhancing the treatment success while decreasing morbidity.^1^ Commonly PFCs resolve completely by themselves, but in few cases can remain, enlarge or becomes infected hence requiring intervention and are named variably according the course they take.^2^

Pancreatic pseudocyst (PPC) is one of the type of fluid collections and has been defined in the revised Atlanta classification as a well-circumscribed, usually round or oval, homogeneous fluid collection surrounded by a well-defined wall with no associated tissue necrosis within the fluid collection and is seen more than four weeks after onset of interstitial edematous pancreatitis.^3^ PPC share a spectrum of clinical features and complications; from being totally asymptomatic to having multiple dreaded complications.^4^ Previously PPC have been dealt by transabdominal route or surgically, but with the advent of EUS this has been modified. EUS in the last 30 years has evolved into an unparalleled modality as far as PPC is concerned.^5^ Initially endoscopic drainages was done without EUS guidance, but being a blind procedure and only dependent on the bulge in the wall of the gut, accompanied with an augmented complication rates and costs, nowadays drainage under EUS guidance has been a preference.^6^ Technically Endoscopic ultrasound (EUS)-guided drainage has many advantages e.g. evaluation of wall thickness, intervening organs, significant blood vessel along the needle path in the cyst wall as well as debris in the cyst can be assessed with great accuracy. Clinically it has high clinical efficacy comparable to surgical or percutaneous approaches, but with minimal morbidity and costs.^7^ EUS is still in the evolution phase and this is perhaps the first study of its type from this part of the world. We did this analysis in order to evaluate the clinical features, radiological findings, technical features, complications and success related to this procedure.

## METHODS

The study was conducted on the patients with symptomatic PPC referred to us for EUS guided drainage over a period of three years, between January 2015 and January 2019, at the surgical Ward-4, Civil Hospital Karachi after the approval of Internal Review Board (Ref. No IRB-1120/DUHS/Approval/2018/, dated October 22, 2018). All the patients, pediatric or adult having:

- Symptomatic PPC’s more than 6 cm in size and more than 4 weeks’ old.
- Compressing symptoms.
- Communication with pancreatic duct on MRCP/ERP.
- Liquefied contents, well-formed fibrous wall and good accessibility were included.

Exclusion criteria comprising all the pregnant patients, walled off necrosis, pseudo aneurysm in the wall, cystic tumors, small asymptomatic pseudocysts (<5 cm) or complicated pseudocysts (multiloculated), presence of large intervening vessels on Power Doppler or absence of a clear access to the pseudo cyst content distance higher than 10mm), severe coagulopathies, uncorrectable severe platelet dysfunction and failure to provide informed consent. The results of all the procedures were analyzed retrospectively and medical records of all the patients with EUS guided drainage were assessed.

For the purpose of this review, clinical records were evaluated according to age, gender, previous surgery, medications used, signs and symptoms, indications, laboratory tests and imaging modalities used, total procedure time, mode of therapy and complications. All the procedures were performed by an expert endoscopists who has performed more than thousand EUS procedures. Written and informed consent was obtained from all the patients and the study was approved by our institutional review board. Propofol was given for deep sedation by the anesthesia specialist for all the procedures. Intravenous cephalosporin was imperative for the procedure and was continued in an oral form for five days. High quality multi slice CT scan was essential in all the patients. Ionic contrast medium Urograffin (A mixture of salts of diatrizoic acid) was used to opacify the cyst, if required. During EUS, arterial oxygen saturation was continuously monitored by a pulse oximeter. EUS was performed by employing a standard technique, using Linear Scanning Ultrasound Endoscope (GF-UCT180; Olympus America Inc.). Scope was placed in the stomach or duodenum and cyst localized with ultrasonography. Cyst was punctured with a 19 gauge Expect™ Endoscopic Ultrasound Aspiration Needle and 0.035 wire passed inside and looped. Puncture was done perpendicular the bowel wall. Initial aspirate of few millilitre fluid was sent for routine biochemical, cytological and microbiological examination Tract was accessed with a Cystotome 8.5-Fr (CST-10, Cook Endoscopy, Winston-salem, NC) and dilated with CRE™ PRO single-use wire guided biliary balloon dilatation catheter (4mm to 6mm) or both. Single or multiple Advanix Double Pigtail stent (7-Fr or 10-Fr) under EUS or fluoroscopic guidance we replaced into the cyst cavity after placing single or multiple guide wires and their position checked. Technical success of the transmural procedure was defined as accessing and successfully placing double pigtail stent/stents within the pseudocyst.

Clinical success was defined as clinical improvement or cyst resolution or significant volume reduction of cyst size which was measured by CT or ultrasound after one month and three months. Recurrence was defined as accumulation of fluid in a previously drained PPC after initial drainage, confirmed on CT scan or abdominal ultrasound. Reintervention was defined as EUS guided transmural drainage after confirmation of recurrence. If the patient develops post procedure bleeding, perforation, infection, pulmonary complication or a pancreatico-pleural fistula within 24 hours or after 30 days’ assessment; they were all defined as post procedure complication. Statistical analysis included mean with standard deviation and median with interquartile ranges for continuous variables. Frequency analysis included percentages for categorical variables. Data was analyzed using SPSS version 17(SPSS Inc., Chicago, IL, USA).

## RESULTS

Our endoscopy database revealed 71 patients of EUS guided pseudocyst drainage. Mean age was 37.20 ± 17.27 years with a range of 6 to 68 years; there were 49 (61.3%) male and 22 (27.5%) female. Localization of the cysts is depicted in Table-I. Median PFC diameter was 10 cm [Interquartile range (8-11 cm)]. Symptoms indicating drainage were abdominal distension (n=36, 50.7%) abdominal pain (n=21, 29.6%), enlarging cyst (3=6, 4.2%), gastric outlet obstruction (n=11, 15.5%). Median white blood cell count was 8 per micro liter [Interquartile range (8-9 per microliter)], median serum albumin was 34 g/liter [Interquartile range (33- 35 g/liter)]. Acute pancreatitis was seen in 52 (73.2%) and 19 (26.8) had chronic pancreatitis. Etiology of pancreatitis was acute gall stone pancreatitis in 37(52.1%), idiopathic in 18 (25.4%), post trauma in 6(8.5%), alcohol in 5(7%) and post ERCP in 5(7%). Total number of daycare cases were 50(70.4%) and 21(29.6%) admitted with a mean admission time of 3.48 days and arrange of one to six days. Mean time of acute episode to drainage was 60.55 ± 18.56 days. Total number of procedures done were 86; one procedure was done in 59, two procedures were done in 9 and three procedures were done in 3. Mean procedure time was 24.15 ± 6.51 minutes. Site of PPC drainage is showed in Table-II. Total number of stents placed by us were 145; one in three, two in 62 and three in six. There were five recurrences in our patients. All patients had a redo procedure and pseudocysts were resolved in all; three had two procedures and two had three. Endosonographic. Features were; anechoic in 57(80.3%), hypoechoic in 14(19.7%), 66(93%) had a bulge in the wall, wall thickness of>1cm in 18(25.4%), debris in 24(33.8%) and septa in 18(25.4%). Aspirated fluid was clear in 41(57.7%), brownish in 17(23.9%) and purulent in 13(18.3%). Median procedure time was 23 ± 13 min. Complications occurred in 12 (8.52%) patients, including stent migration (5/12), bleeding (4/12), infection (1/12), intra-abdominal abscess (1/12) and perforation (1/12). Three of the stents were displaced distally and were re-stented and had an uneventful recovery. In two patients, stents were displaced inside the cyst, both the patients were re-stented because of infection and lots of pus came out. Once the patients were stabilized stents were removed endoscopically and both of them had an uneventful recovery. Three patients had a minor bleed from the puncture sites which resolved spontaneously, one patient had a major bleed and was recovered after angiographic embolization. One patient had a secondary infection, both the stents were blocked and the patient was recovered after triple stenting of the cyst. One patient had a perforation and another one had an intra-abdominal abscess both of them were referred for surgery. Technical success was achieved in all (100%) and clinical success in 97.1%. There was no procedure-related mortality.

**Table-I:** Anatomical site of pancreatic pseudocyst.

Site	Frequency	Percentage
Body & Tail	33	46.5%
Tail	19	26.8%
Head	17	23.9%
Body	2	2.8%

**Table-II:** Site of PPC drainage.

Site	Frequency	Percentage
Stomach	69	97.2%
Duodenum	2	2.8%

## DISCUSSION

For symptomatic PPC, EUS guided drainage has become the modality of choice as compared to the surgical or percutaneous approach, due to its technical superiority as well as amelioration in patient related factors.^8^ PPCs can be drained endoscopically with the help of a single or multiple plastic stents or fully covered self-expanding lumen apposing metal stent (LAMS).^9^ LAMS are good in draining PPC but comes with a hefty price tag. Our unit is a tertiary care center providing free of cost treatment to our patients, we used plastic stents in all our patients. New studies have been published claiming no difference in clinical outcomes with plastic or LAMS, plastic stents decreased the cost of procedure but LAMS were associated with shorter procedure time.^10^ We used single or multiple double pigtail stents in all the cases with varying sizes due to its low rate of complications especially erosion of a vessel associated with straight stents. Average time consumed in our study was 24 minutes, this was mainly due to expertise of our assistants.

Our technical success rate of EUS guided pseudocyst drainage was 100% which was comparable to other series.^11^-^13^ We used cystotome to access the PPC with minimal complications. We used fluoroscope for locating the scope and used cystotome to dilate the tract in all the cases.^14^ There is a technical ease with cystotome and low risk of complications. We placed two wires from within the cystotome in the cyst cavity after dilatation, making the stent placement easier. Different groups have tried variety of different maneuvers and instruments to facilitate the access of the PPC. Rana et al. showed non-fluoroscopic endoscopic ultrasound-guided transmural drainage of pseudocysts.^15^ One group used cystotome without fluoroscope with a success rate of 83.3% as well as using Giovannini needle with a success rate of 94%.^15^,^16^ Newer devices are coming in the market, all are associated with LAMS, with good success rates.^9^ Our short term clinical response was 92%, comparable to the study by Yang D et al.^17^

Complications occurred in twelve patients, with bleeding being the most common. Secondary infection was the second most common complication. We don’t know it was the stent size or the number of stents being used. According to some authors, multiple stents or large diameter stents are helpful for the drainage of fluid collection and solid debris evacuation, but along with that they might increase the back flow of bacteria from the gastric cavity thus increasing the chances of secondary infection.^18^ One patient had a stent displaced in the cyst, which was removed with the help of pediatric scope in two sessions because of secondary infection of the cyst. EUS guided drainage has lower rate of complications as compared to conventional transmural drainage as shown in many studies.^19^,^20^ Although our study was not a comparative study but low rate of complications infers the same conclusions.

Total number of stents placed by us were 145, in all the patients with a mean number of two stents. The resolution rate was found to be 97.1% at the end of follow up. Saftoiu et al. reported a symptomatic relapse rate of 8%^21^, our relapse rate was 7%, which may be due to the use of plastic stents. There were children and adolescent in our series with a success rate comparable to adult patients. Multiple studies have proved that EUS guided procedure is safe in children and should be the first line of therapy when indicated.^22^

In our series, we did not perform any transpapillary pancreatic duct stenting. Multiple studies have proven that there is no added advantage of endoscopic retrograde pancreatography (ERP).^23^ Our results are comparable to the other series signifying that ERP is not essential in the treatment plan and can prone the patients to complications related to ERP specially pancreatitis.

## CONCLUSION

Pancreatic pseudocyst (PPC) is a known complication of acute as well as chronic pancreatitis which can have dreaded and appalling effects. Different modalities are available for the treatment of PPC with different complications and morbidities. In this part of the world with limited and scarce resources, EUS guided drainage of PPC is most feasible and rational with minimal complications, thus making it a front runner procedure. Every endoscopist should be well versed with the complications related to this procedure and every effort should be done to minimize it.

### Authors’ Contribution

**EK** conceived, designed, did statistical analysis, editing of manuscript and is responsible for integrity of research.

**MAT, IZ & JAK** did data collection and manuscript writing.
